# SA Rugby Injury and Illness Surveillance and Prevention Project (SARIISPP): The Carling Currie Cup Premiership Competition, Injury Surveillance Report, 2021

**DOI:** 10.17159/2078-516X/2022/v34i1a15259

**Published:** 2022-01-01

## Executive Summary

As part of the South African Rugby Injury and Illness Surveillance and Prevention Project (SARIISPP), the annual Carling Currie Cup 2021 Premiership Division Competition (‘Carling Currie Cup’) injury data are recorded by the medical doctors and medical support staff of the respective teams. SARIISPP has been collecting and analysing these data annually since 2014 for the Carling Currie Cup tournament. All seven teams are required to record injuries that occur in each match and training session in the team throughout the season. Additionally, the strength and conditioning coaches record their training sessions throughout the season for training exposure data.

The analysis shows injury patterns over time between tournaments, teams, and international tournaments. When investigating these patterns, areas of concern are identified. Where appropriate, changes in the game, tournament structure or medical support services are considered or contested against the evidence. Also, injury specific interventions can be created and implemented, where the evidence indicates such a need.

Throughout this report, injury burden and injury rate are used for analysis. Although teams may have a low injury rate, injuries of a high severity still represent a sizable burden to the team, resulting in many training and match days lost due to injury for that team. This highlights the importance of collecting severity data, and not simply injury rates on their own.

The injury rates are expressed as the mean (95% confidence interval) per 1000 player hours. The injury rate of Time-Loss injuries for the Carling Currie Cup 2021 was 89 (74 to 104) injuries per 1000 player hours which is similar to the international rate of 91 (77 to 106) injuries per 1000 player hours [1], and within the expected limits of season-to-season variation for the Carling Currie Cup. This equates to 1.8 injuries per team per match and an injury burden of 1935 days lost per 1000 player hours.

The Toyota Free State Cheetahs had the highest injury rate for Time-loss injuries throughout the Carling Currie Cup 2021 tournament. The Airlink Pumas had a significantly lower injury rate in 2021 in comparison to their 2014/15–2020/21 tournament average. Despite having the highest injury rate, the Toyota Free State Cheetahs had one of the lowest average severities of 17 days absent per injury. This means that although the Toyota Free State Cheetahs had a high number of injuries, they did not lose many days of training and match play due to these injuries. In 2019, it was found that during previous seasons the teams ranked 1st or 2nd in the competition had significantly lower injury rates than those who ranked in last position [2]. However, in 2021 the Vodacom Blue Bulls who won the tournament, had a moderate injury rate and average severity, resulting in them experiencing a moderate injury burden as a team. The Sigma Golden Lions, who placed last in the tournament had one of the lowest injury rates and injury burden.

The average severity of Time-Loss injuries in the 2021 tournament was 22 days, which is lower than the 27 days reported in the international meta-analysis [1]. The median injury severity of all Time-Loss injuries was 10 days, with 25% of injuries lasting 6 days or less and 25% of injuries lasting 23 days or more due to injury.

Sprain/Ligament was the most common injury throughout the 2021 Carling Currie Cup tournament, with muscle (rupture/strain/tear) and Contusion/Bruise injuries recording the second and third most common injury types, respectively. The head, knee and ankle were the most injured body locations. Ankle injuries almost doubled in injury incidence and number since the 2020/21 season. The concussion number has seemingly stabilized over the last two years.

In 2021, SARIISPP’s software developers upgraded their data collection software. Unfortunately, the *Injury event* variable could not be captured correctly in this upgraded software package. In some cases, the *Injury event* could be back engineered from the raw data, or was sourced retrospectively from the medical staff, but many cases remained where the medical staff and authors did not want to presume the *Injury event* incorrectly. Therefore, in this report, those *Injury event* cases were recorded as ‘Not provided’. As a result, the *Injury event* data, and any changes in *Injury event* data over time for 2021 must be interpreted with caution. This has been addressed with the software developers for future research. Keeping this in mind, *being tackled* accounted for the highest proportion of injuries in the 2021 tournament, 16% of overall injuries.

A total of 59 Time-Loss training injuries were sustained in the Carling Currie Cup 2021, meaning that 30% of all Time-Loss injuries occurred during training. This equates to an incidence of 2 (1 to 2) injuries per 1000 training hours and is lower than the meta-analysis injury incidence of 3 (2 to 4) injuries per 1000 training hours (1). The average severity of Time-Loss training injuries was 42 days absent.[Fig f20-2078-516x-34-v34i1a15259][Fig f21-2078-516x-34-v34i1a15259]

**Figure f20-2078-516x-34-v34i1a15259:**
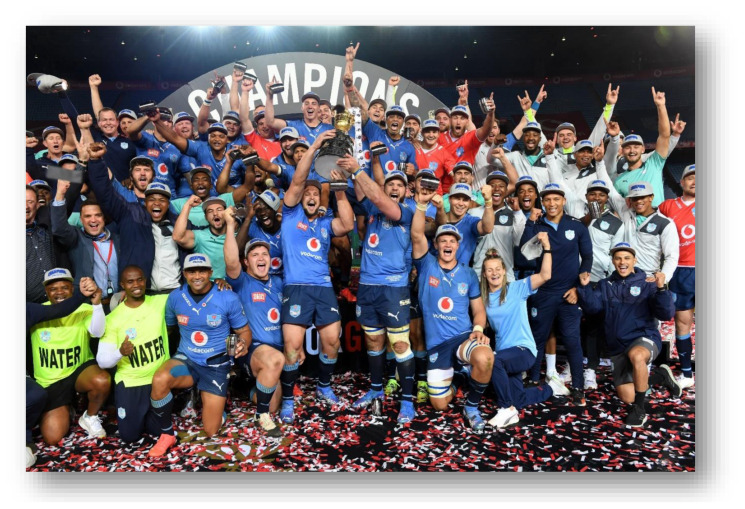


**Figure f21-2078-516x-34-v34i1a15259:**
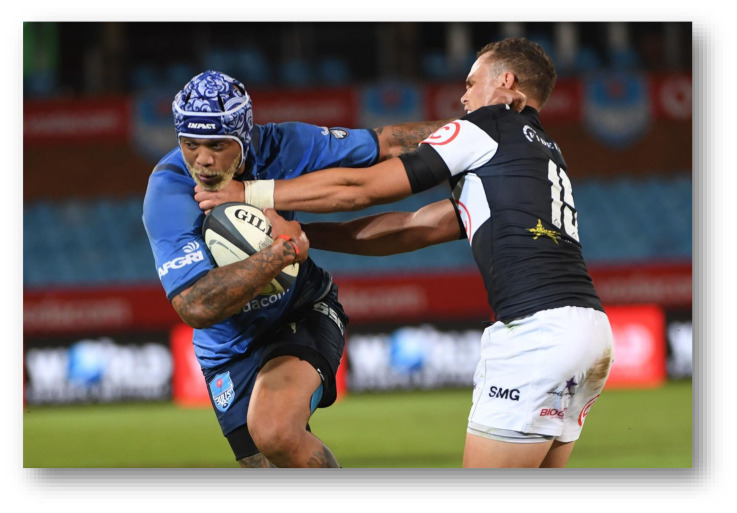


## Introduction

In 2014, as part of the SA Rugby Injury and Illness Surveillance and Prevention Project (SARIISPP), the South African Rugby Union (SA Rugby) implemented a new standardised injury surveillance format for the Carling Currie Cup Premiership Division Competition. This required the team doctor or medical support staff to record all relevant match and training injury data according to the standardised *BokSmart* injury surveillance data capture format. The definitions and format of reporting is aligned with the 2019 IOC consensus statement for injury recording in sport [4], and for rugby union [3].

Injury surveillance is an essential step in injury prevention. Specifically, injury surveillance is important for developing injury prevention strategies, and then testing their efficacy and effectiveness after implementation. When Injury surveillance is captured in the standardised format it enables comparison of injury rates between teams within the same tournament, tournament injuries over successive years, and with other rugby injury surveillance studies.

Reports on rugby tournament injuries present the injury numbers as a rate (or incidence) i.e., the total number of injuries divided by the total amount of time exposed to the risk of experiencing an injury. The standardised format is to present the number of injuries per 1000 player exposure hours. Match exposure hours are calculated as the number of matches played multiplied by the number of exposed players (15) and the match exposure time (80 mins). Training exposure hours are calculated as the average number of players present at training multiplied by the average time spent training each week; this is then summed to calculate training exposure hours over the competition period. As discussed, throughout this report the standardised injury rates have been provided to allow for comparison with other reports. Every effort has been made to present these rates on a ‘per team’ and ‘per match’ level for easier and more pragmatic interpretation.

Since 2016, the Carling Currie Cup medical doctors and medical support staff were asked to record the physical return to play date of the injured players, thereby allowing for the actual severity of the injury to be calculated. Injury burden is a combination of the injury rate and severity and is expressed as the number of days absent from training and matches per 1000 player hours. Throughout this report only actual, rather than predicted severity is used for analysis.

In the report, the 2014 and 2015 season data are included in the sections reporting on injury numbers and incidence only. The sections reporting on injury severity and burden start from the 2016 season when the severity data was first collected.

During the Carling Currie Cup 2020/21 seasonal report, Time-Loss training injury and training exposure data started being captured as a part of the South African Rugby Injury and Illness Surveillance and Prevention Project (SARIISPP). The addition of training exposure and injury information to the SARIISPP data collection enables better interpretation of the injury data.

An inherent bias with most injury surveillance studies is that the teams’ medical doctors or medical support staff are exclusively responsible for entering their team’s injury data. As no audit process is done on the collection of these data, in many of these cases, the accuracy of the data is dependent on the compliance of the doctors or medical support staff. This potential limitation is present in most injury surveillance studies. To minimise this potential limitation, SARIISPP had a project coordinator who was in frequent contact with the medical doctors or medical support staff to ensure they were up to date with the data capturing.

The Carling Currie Cup 2021 semi-finals were contested between DHL Western Province vs. Cell C Sharks and Vodacom Blue Bulls vs. Xerox Golden Lions. The final was between Vodacom Blue Bulls vs. Cell C Sharks, with the Vodacom Blue Bulls eventually winning the tournament for the second consecutive season.[Fig f22-2078-516x-34-v34i1a15259]

**Figure f22-2078-516x-34-v34i1a15259:**
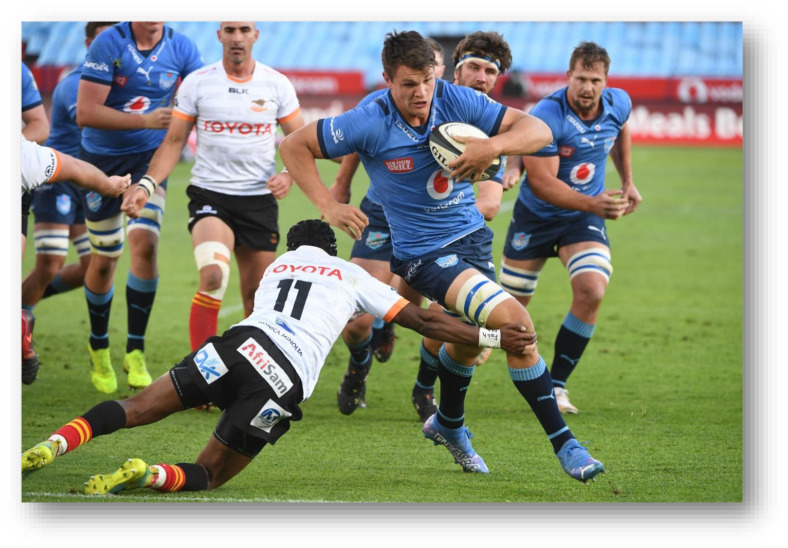


## Definitions

All definitions are originally based on the 2007 consensus statement for injury reporting in rugby union [3] and have since been realigned with the latest International Olympic Committee (IOC) consensus statement for methods of recording and reporting epidemiological data on injury and illness in sport [4].

### MEDICAL ATTENTION INJURY

All injuries that were seen by the teams’ medical doctor or medical support staff were classified as Medical Attention injuries. These injuries are defined by the 2007 statement as an “*injury that results in a player receiving medical attention”* [3], and by the more recent IOC statement as *“a health problem that results in an athlete receiving medical attention”* [4].

### TIME-LOSS INJURY

Medical Attention injuries were further categorised as Time-Loss injuries, where appropriate, and defined by the 2007 statement as, “*an injury that results in a player being unable to take a full part in future rugby training or match play*” [3]. The IOC definition is, *“a health problem that results in a player being unable to complete the current or future training session or competition”* [4].

### INJURY RATE

For this report, an injury rate is the number of injuries expressed per 1000 player exposure hours. This method of expressing injury rate has been used in previous years’ reports of the Carling Currie Cup Premiership tournament and other international literature, and therefore makes comparisons easy. Moreover, the injury rate is expressed as a mean with 95% confidence intervals. A 95% confidence interval around a mean value indicates that there is a 95% chance (i.e., very high chance) that the true value falls within this range. In this report, we present the 95% confidence intervals assuming normal distribution of the data and use the approach of examining the overlap of the confidence intervals, to determine whether the injury incidences are significantly different; if the range of confidence interval values of two comparisons do not overlap, there is a strong chance (95%) that their injury rates are different from each other. We have opted for this method because it is easy to use, conservative and less likely to produce false positive results [5].

### MEDIAN (INTERQUARTILE RANGE)

When numbers are ordered from the lowest to highest, the median is the value which separates the higher half of the values from the lower half of the values. Simply put, it is the middle value of a list of ranked numbers. The interquartile range (IQR) describes the spread of the data. When rank ordered data are divided into quartiles the first and the third quartile represents the value under which 25% and 75% of the data points fall, respectively. As an example, a team may have a median injury severity of 32 days (IQR 7 to 40). This means that when the teams’ injury severities are rank ordered the mid-point or median of the injury severities is 32 days. Also 25% of their injuries result in 7 or less days absent from training and matches and 25% of their injuries result in 40 days or more absent from training and matches.

### NEW, SUBSEQUENT AND RECURRENT INJURIES

In 2019, in the Carling Currie Cup Premiership Division Competition, a ‘*New Injury’* was defined as when a player sustained his first injury. Any injury that the *same* player sustained after this initial injury was defined as a *‘Subsequent Injury’.*

According to the more recent IOC statement, any subsequent injury to the same site and of the same type is referred to as a ‘*Recurrence’* if the index injury was fully recovered before reinjury, and as an *‘Exacerbation’* if the index injury was not yet fully recovered [4].

To provide more detail on the subsequent injuries for practitioners, we have further categorized the subsequent injuries in this report into one of four groups based on the OSICS classification diagnosis:

- Different site - Different type
- Different site - Same type
- Same site - Different type
- Same site - Same type

According to the 2007 Consensus Statement for rugby, any subsequent injury classified as ‘Same site - Same type’ was a *‘Recurrent injury’* [3].

### INJURY SEVERITY

The total severity of an injury is defined as *“the number of days that have elapsed from the date of injury to the date of the player’s return to full participation in team training and availability for match selection”* [3,4]. The actual severity of each injury is classified by the severity groupings provided in the 2007 consensus statement; *Slight* (0–1 days lost), *Minimal* (2–3 days lost), *Mild* (4–7 days lost), *Moderate* (8–28 days lost), *Severe* (>28 days lost), *Career ending* and *Non-fatal catastrophic* [3]. To align with the latest IOC statement the injuries have been re-grouped to reflect the severity groupings *‘1–7 days’, ‘8–28 days’ and ‘>28 days’* [4].

The average severity represents the average number of days lost per injury when dividing the accumulated total number of days lost by the total number of injury events. For example, a team may have a total severity of 550 days absent, accumulated from 22 injuries. The average severity of the team’s injuries would therefore be 550/22, which equals, on average 25 days absent per injury.

### INJURY BURDEN

Injury burden is a combination of injury rate and severity. It is the injury rate multiplied by the average severity (number of days lost due to injury) and is expressed as the number of days absent per 1000 player hours. For example, a team who has an injury rate of 75 injuries per 1000 player exposure hours, and an average severity of 38 days lost per injury will have an injury burden of 2850 days absent per 1000 player hours (i.e., 75 × 38 = 2850).

### OPERATIONAL INJURY BURDEN

The operational burden is the expected number of days lost per injury per team for every match played over the tournament or season. The measure is an extrapolation of injury rates and severities over a season and includes the most severe injuries together with the least severe injuries in its estimation. For example, if a team has an operational injury burden of 2 days, it means that based on their injury rates and average severity, on average, 2 days absence can be expected from every match injury the team sustains.

### META-ANALYSIS

A meta-analysis is a study using statistical methods to combine multiple scientific studies with varying levels of evidence on the same topic to determine overall defining patterns and results from the combined data. As such, it represents the highest level of scientific evidence available. The findings in this report are compared to the data in the most recent meta-analysis, published in 2021, for rugby union injuries at an elite professional level [1].[Fig f23-2078-516x-34-v34i1a15259]

**Figure f23-2078-516x-34-v34i1a15259:**
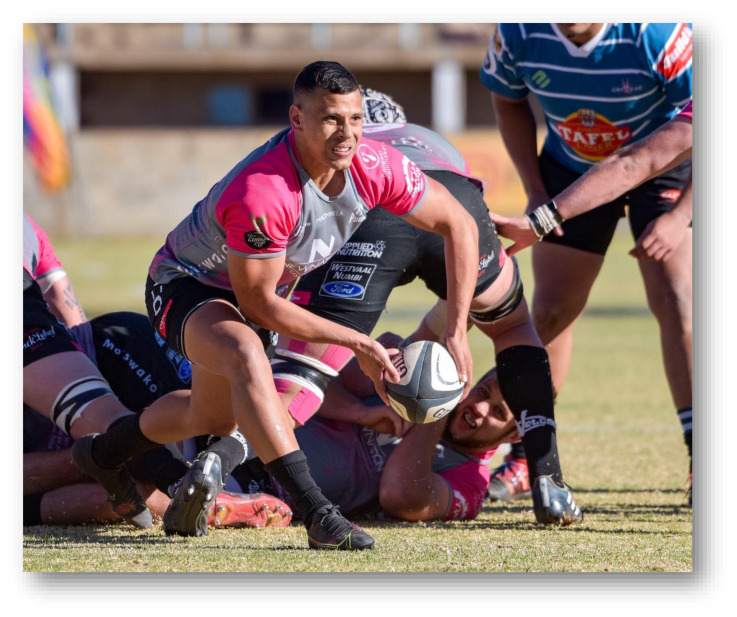


## MATCH INJURIES

### Injured players

During the Carling Currie Cup 2021, 96 players sustained a total of 135 Time-Loss injuries. We cannot account for every player who entered or left the match day squads, either as an injury replacement or for other reasons. We have therefore, assumed a total of 161 players were exposed to playing rugby matches in the tournament (7 teams × 23 players per match-day squad). Sixty percent (60%) of the 161 players sustained a match injury during the tournament (Figure 1a). The proportion of players who sustained one Time-loss injury increased slightly from 2020/21 to 2021. Furthermore, the proportion of players who experienced 2 or 3 injuries increased from 2020/21 to 2021 (Figure 1b). Only the absolute number of Time-loss injuries will be analysed in this report, regardless of the number of players who sustained them.

### Overall Injury Rate

Only Time-loss injuries have been analysed because these injuries are more comparable between different teams, tournaments and with the published scientific literature [1]. As mentioned previously, Time-loss injuries resulted in players missing a match or training session.

**Figure 2:**
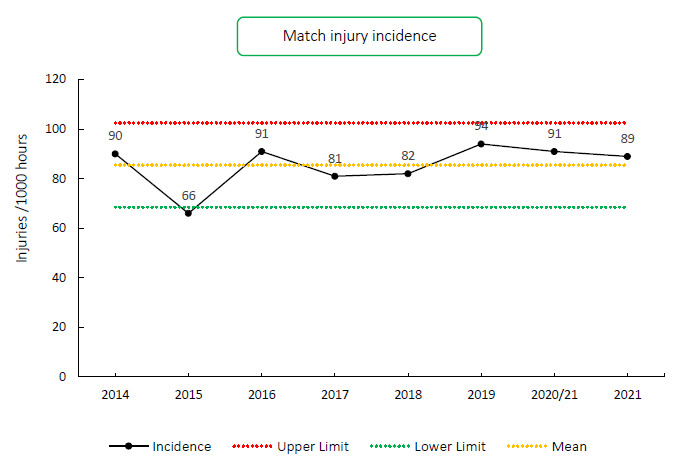
Injury incidence of Time-Loss match injuries over the surveillance period with mean ± standard deviations shown.

The overall match injury incidence for the Carling Currie Cup 2021 was 89 (74 to 104) injuries per 1000 player exposure hours. This is similar with the injury incidence of the meta-analysis of 91 (77 to 106) injuries per 1000 player hours [1] and is within the expected limits of season-to-season variation for the Carling Currie Cup tournaments based on the last 7 years (Figure 2). An injury incidence of 89 injuries per 1000 player hours equates to 1.8 injuries per team per match.

**Figure 3:**
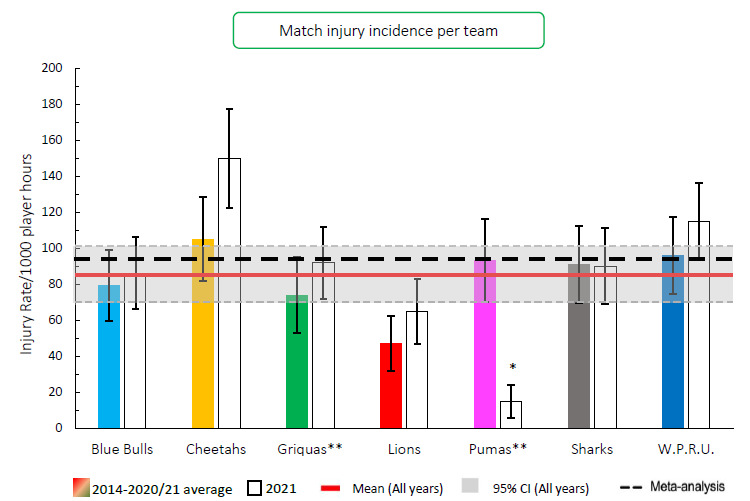
Injury incidence for Time-Loss injuries experienced by each team in the Carling Currie Cup 2021 in comparison to their 2014–2020/21 averaged injury rate. (**) Average injury rates for Pumas 2015 – 2020/21 and Griquas for 2015, 2016, 2018, 2019 and 2020/21. Asterisk (*) indicates that a team’s 2021 injury rate is significantly different to their 2014–2020/21 averaged injury rate.

When comparing the team’s 2014–2020/21 averaged tournament injury incidence to their 2021 season’s injury incidence, the Airlink Pumas experienced a significantly lower injury incidence rate in 2021 in comparison to their 2014/15–2020/21 tournament average (Figure 3). No team showed significantly higher injury incidences compared to their 2014–2020/21 tournament averages.

Overall, the combined average injury incidence of 85 (70 to 101) injuries per 1000 player hours for all the teams over the last 8 years is similar to the international meta-analysis summary of 91 (77 to 106) injuries per 1000 player hours [1].[Fig f24-2078-516x-34-v34i1a15259]

**Figure f24-2078-516x-34-v34i1a15259:**
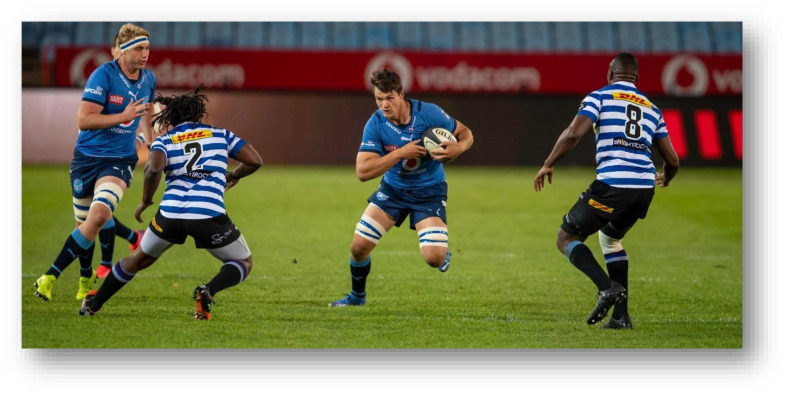


### Injury incidence over the season

**Figure 4:**
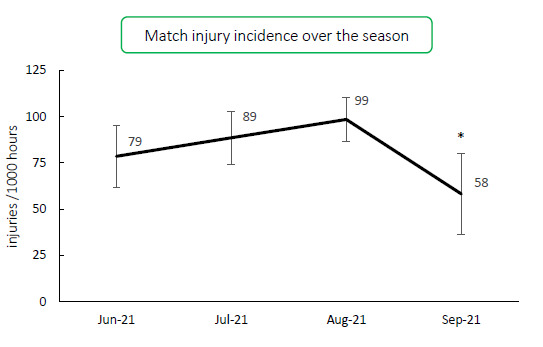
Match injury incidence per month of the 2021 Currie Cup season. Asterisk (*) indicates that the injury incidence is significantly lower in September than in August.

When looking at the Time-loss injury incidence over the 2021 Carling Currie Cup tournament, the injury incidence in September was significantly lower than in August. Throughout the other months in the 2021 season, there were no significant differences (Figure 4).

### Overall Severity

**Figure 5:**
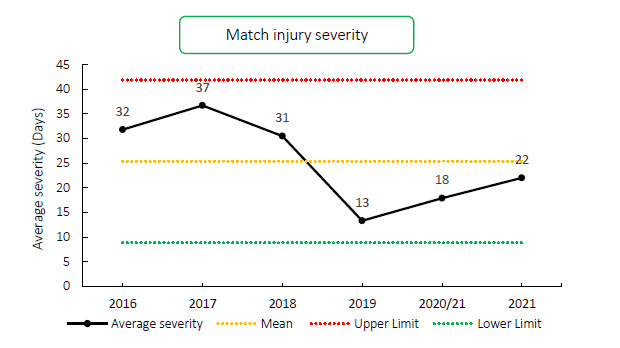
Mean severity of Time-Loss match injuries over the surveillance period with mean ± standard deviations shown.

The average severity of match injuries for the Carling Currie Cup 2021 was 22 days, which is lower than the average for the Currie Cup tournament 2016–2020/21 (25 days) but was within the expected limits of season-to-season variation (Figure 5). The median severity was 10 days (IQR 6 to 23). This means that the half-way mark of the injury severities was 10 days, with 25% of all Time-Loss injuries lasting for 6 days or less and 25% lasting 23 days or longer. The 2021 median severity is higher than in 2019 and 2020/21 (4 (IQR 3 to 10) and 4 (IQR 1 to 12), respectively) but similar to 2018 which had a median severity of 11 days (IQR 5 to 35). Therefore, the increase in severity in 2021 could be because of more severe injuries and does not seem to be a trend currently.

When the medical doctors or medical support staff clinically assessed the injured player, they recorded the injury time from the starting date. Similarly, when the player returned to play, the return to play date was recorded. From these two dates, the injury severity is determined.

These data are grouped to align with the latest IOC statement. The severity groupings include *‘1–7 days’, ‘8–28 days’ and ‘>28 days’* [4].

**Figure 6:**
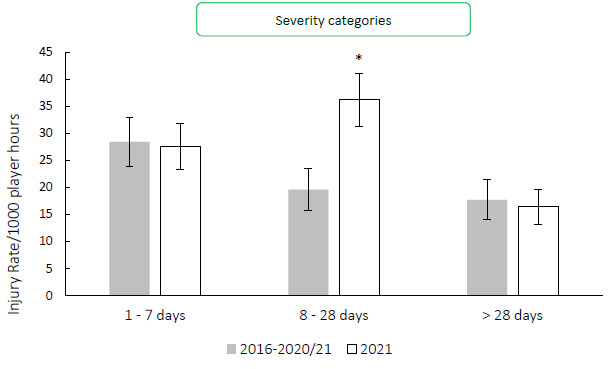
The actual severity category injury rates for the Carling Currie Cup 2021 in comparison to the averaged injury rates for the 2016–2020/21 actual severity categories.

Figure 6 compares the injury severity rates for the Carling Currie Cup 2021 tournament to the average of the 2016–2020/21 tournament averaged rates. The severity category of ‘*8–28 days’* was significantly higher in 2021 in comparison to its 2016–2020/21 average (Figure 6).[Fig f25-2078-516x-34-v34i1a15259]

**Figure f25-2078-516x-34-v34i1a15259:**
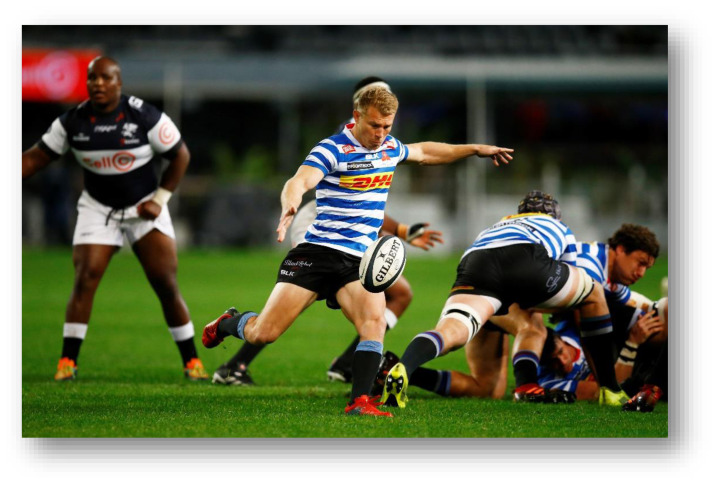


Table 1 describes the actual severity of each teams’ Time-Loss injuries for the Carling Currie Cup 2021. The Vodacom Blue Bulls have again been used as a worked example to explain Table 1. The Vodacom Blue Bulls sustained 1.7 injuries per match, meaning that for every 0.6 matches played they sustained one injury. In total, the Vodacom Blue Bulls lost 453 training and match days due to injury. This equates to an average of 24 training and match days lost for every injury sustained. The burden of the team’s injuries equates to 2060 days lost per 1000 player hours. Translating this to an operational burden per match, it shows that the Vodacom Blue Bulls lost 41.2 days per injury per match over the season. The median injury severity for the Vodacom Blue Bulls was 10 days (IQR 6 to 34). This means that when severities of the Vodacom Blue Bulls Time-Loss injuries were rank ordered, the midpoint of the severities was 10 days off from rugby, with 25% of their injuries lasting equal to or less than 6 days off and 25% of their injuries lasting equal to or longer than 34 days off.

**Figure 7:**
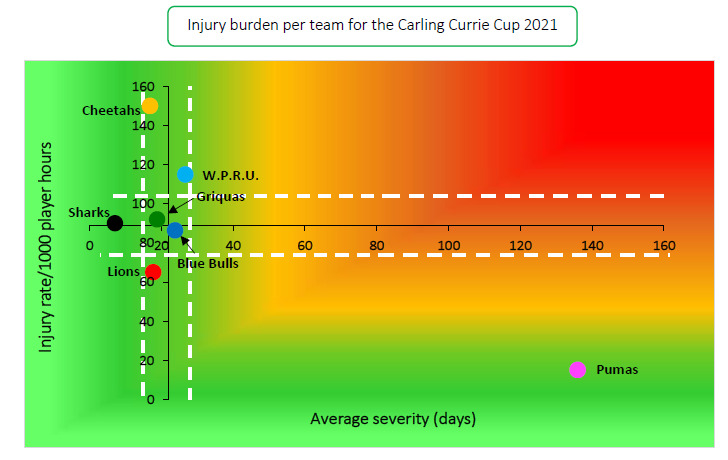
Injury rate plotted against the average severity of Time-Loss injuries for each participating team in the Carling Currie Cup 2021. The Y-axis Average Injury Rate is expressed as the tournament average (±95% CI) and X-axis Average Severity is expressed as the average (±95% CI) of the individual injury severities in the tournament.

The Toyota Free State Cheetahs had the highest rate of Time-Loss injuries, but these were of low severity. In contrast, the Airlink Pumas by far had the lowest injury rate, but their injuries were of the highest severity (Table 1; Figure 7). Teams who fall in the green zone (below average and 95%CI), will generally not be impacted as much by their injury burden, regardless of whether their injury rate or average severity is relatively high. As soon as the combination of rate and severity moves into the orange (close to average) and/or red zone (above average and 95% CI), the impact on team performance and player availability becomes more problematic. None of the teams that participated in the 2021 Carling Currie Cup were in the orange or red zones. However, DHL Western Province showed the highest injury burden because of their combination of injury rates and severity.

All the data in this report is aligned with the 2019 IOC consensus statement [4] and is further presented as such to compare against previous season reports and the international meta-analysis [1]. Table 2 presents the Carling Currie Cup 2021 injury data in the format recommended by the 2019 IOC consensus statement. This table provides an overview of the Tissue and Pathology types of injuries sustained during the 2021 season. Furthermore, this format is used throughout this report.

### New, Subsequent and Recurrent Injuries

During the Carling Currie Cup 2021, the overall injury incidence for *New injuries* was 63 (51 to 76) injuries per 1000 player hours. This is a similar injury rate to the Carling Currie Cup 2020/21.

**Figure 8:**
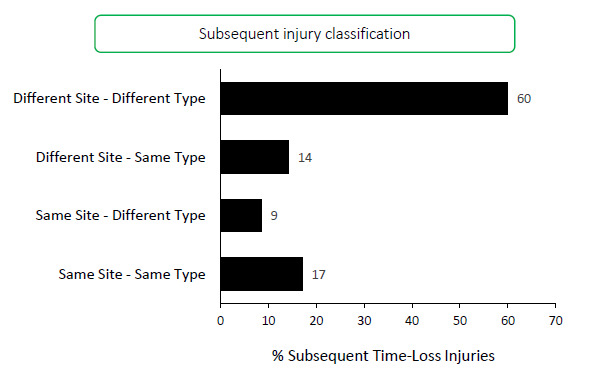
Classification of subsequent injuries for the Carling Currie Cup 2021. Data expressed as a % of subsequent Time-Loss injuries.

Sixty-eight players experienced only one injury during the Carling Currie Cup 2021 season (71% of all injured players). Sixty percent (60%) of subsequent injuries to those 28 players sustaining multiple injury events during the season (Figure 1a), occurred at a different anatomical site and/or were of a different type when compared to the initial index injury. ‘*Different site – different type*’, ‘*different site – same type*’ and ‘*same site – different type*’ are classified as subsequent new injuries. Figure 8 shows the percentage subsequent Time-loss injuries.

A subsequent *recurrent* injury was any subsequent injury classified as ‘*same site – same type*’, which refers to the same location and same tissue type involved as the original index injury. Only six subsequent recurrent injuries occurred in the Carling Currie Cup 2021.

The injury incidence for subsequent recurrent injuries was 4 (1 to 7) injuries per 1000 player hours, which is slightly higher than the 2020/21 tournament’s injury incidence of 3 (0 to 6) injuries per 1000 player hours.

There is a slight increase in both the proportion of new injuries and subsequent *recurrent* injuries compared to the Carling Currie Cup 2020/2021 tournament. The 2021 season has the highest proportion of subsequent *recurrent* injuries across the Carling Currie Cup 2016 – 2021 tournaments (Table 3).

### Injury Type

Overall, Ligament sprain was the most common Time-loss injury during the Carling Currie Cup 2021 (27%), followed by Muscle (rupture/strain/tear) injuries (22%).

The median severity for Ligament sprain injuries was 12 days with 25% of injuries resulting in 9 or less days absent from training and matches, and 25% of injuries resulting in 19 or more days absent from training and matches (Table 4). The average severity was 27 days absent.

**Figure 9:**
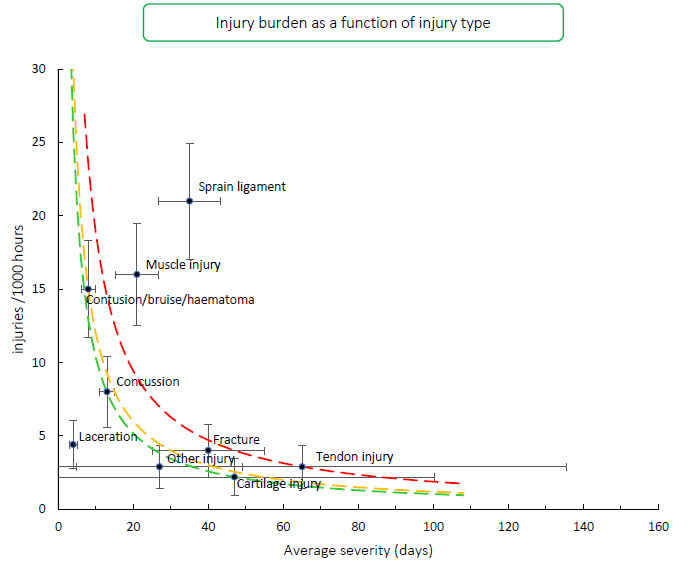
Injury burden as a function of injury type for the seasons 2016 – 2021. The y-axis represents incidence (number of injuries per 1000 hours) and x-axis represents the average severity (days absence) per injury type. Green line: values to the left and below represent those under the 25^th^ burden percentile; these are low-risk injuries. Orange line: values to the left and below represent those under the 50^th^ burden percentile; these include the low-medium risk injuries. Red line: values to the left and below represent those under the 75^th^ burden percentile; these include the medium-high risk injuries. Values to the right and above the red line are the most high-risk types of injuries, and impact players and teams the most.

Figure 9 shows the injury burden for the 2016–2021 seasons. Ligament sprain followed by Muscle injury were the two injury types with the highest burden when injury types were combined from 2016–2021 Carling Currie Cup tournaments. These injury types have the highest combination of injury incidence and average severity of injury. Similarly, to 2020/21’s season, these two injury types dominate through the different teams.

The most common Time-loss injuries during the Carling Currie Cup 2021 tournament were joint (non-bone)/ligament injuries (comprised of dislocation/subluxation and sprain/ligament injuries) at 26 (18 to 35) injuries per 1000 player hours. The average severity of joint (non-bone)/ligament injuries in the Carling Currie Cup 2021 was 27 (16 to 38) days.

Following joint (non-bone)/ligament injuries, muscle/tendon injuries (comprised of muscle rupture/strain/tear, tendon injury/rupture and tendinopathy injuries) were the next most common injury. The injury rate for muscle/tendon injuries was 24 (16 to 31) injuries per 1000 player hours. The average severity for muscle/tendon injuries was 28 (13 to 41) days. The injury incidence for the central nervous system during the Carling Currie Cup 2021 was recorded at 7 (3 to 12) injuries per 1000 player hours.[Fig f26-2078-516x-34-v34i1a15259]

**Figure f26-2078-516x-34-v34i1a15259:**
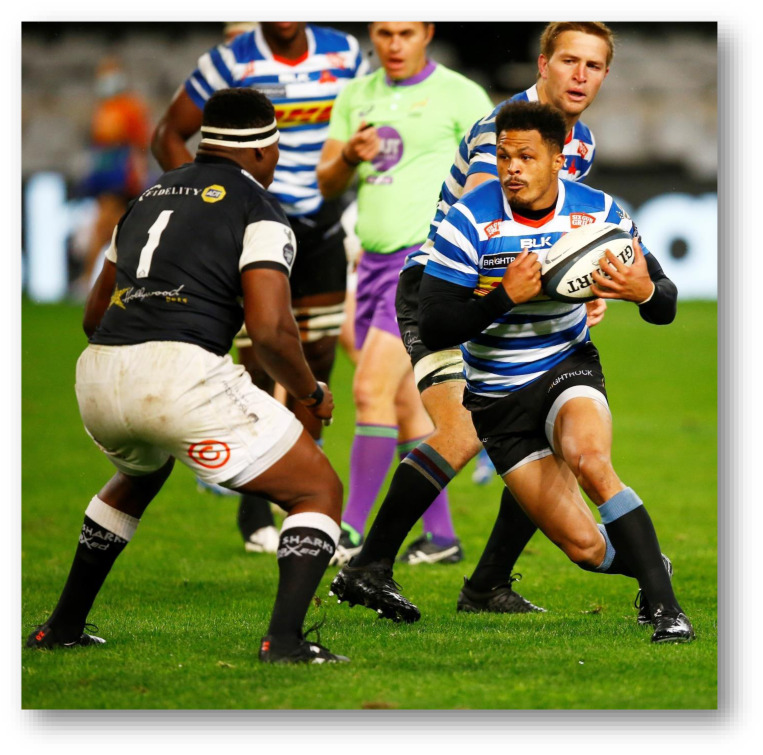


### Injury Diagnosis [6]

The most common Orchard Sports Injury Classification System (OSICS) diagnosis^[7]^ in the Carling Currie Cup 2021 was Concussion (OSICS code = HNCX) followed by Ankle Sprain (AJXX). (Table 5).

## Concussions

**Figure 10:**
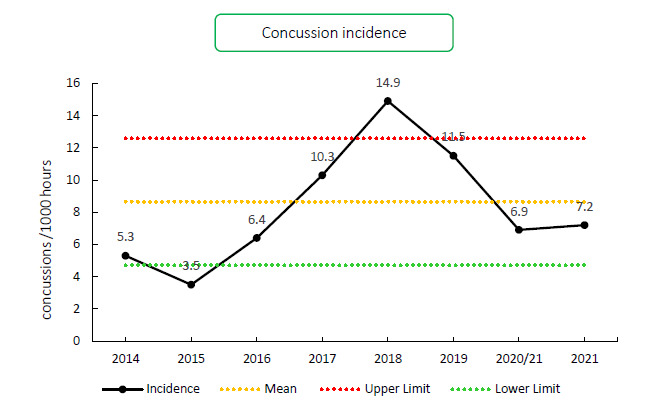
Incidence of concussion over the surveillance period with mean ± standard deviations shown.

Overall, concussions contributed to 11 injuries throughout the Carling Currie Cup 2021 (8%). This equates to 7 (3 to 12) concussions per 1000 player hours. From 2018 to 2020/21, concussion rates decreased. In the Carling Currie Cup 2021 tournament, the injury incidence remained similar to the 2020/21 season: from 6.9 injuries per 1000 player hours in 2020/21 to 7.2 injuries per 1000 player hours in 2021. This still falls below the mean and within the expected limits of season-to-season variation for the Carling Currie Cup (Figure 10). The average severity of concussions reported in the 2021 tournament was 15 days with a median of 12 days (IQR 8 – 18 days). The current South African Rugby concussion regulations do not normally allow for adult players to return within less than 12 days of the concussive event. As this competition takes place at the professional level and is a World Rugby approved tournament, Advanced Care protocols are implemented by the medical practitioner that could potentially allow a player to return-to-play in less than 12 days. These Regulations have recently been amended by World Rugby.

Advanced care clinical settings are defined in the World Rugby and SARU’s Concussion Guideline documents:

- World Rugby Concussion Guideline document - https://playerwelfare.worldrugby.org/
- SARU’s Concussion Guideline documents (When can a player safely return-to-play following a concussion) www.boksmart.com/concussion

In 2021, SARIISPP’s software developers upgraded their data collection software. Unfortunately, the *Injury event* variable could not be captured correctly in this upgraded software package. In some cases, the *Injury event* could be back engineered from the raw data, or was sourced retrospectively from the medical staff, but many cases remained where the medical staff and authors did not want to presume the *Injury event* incorrectly. Therefore, in this report, those *Injury event* cases were recorded as ‘*Not provided*’. As a result, the *Injury event* data, and any changes in *Injury event* data over time for 2021 must be interpreted with caution. This has been addressed with the software developers for future research.

**Figure 11:**
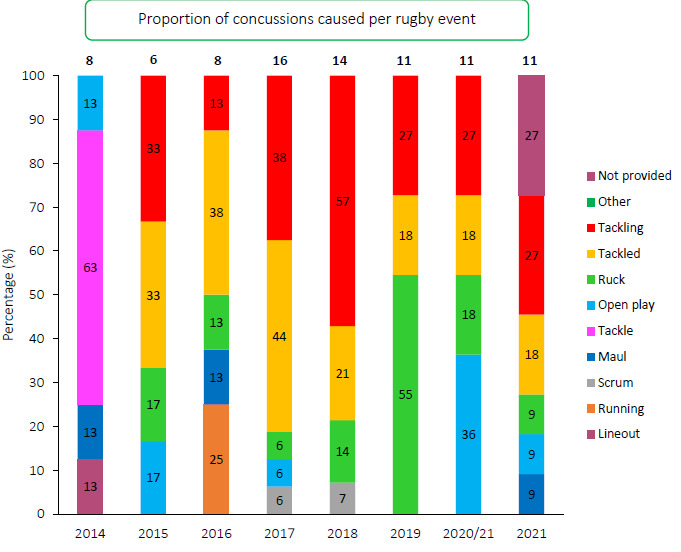
Proportion of concussions caused by the different injury events from 2014 to 2021. (The number above each bar represents the total number of concussions for that year).

Figure 11 shows the proportion of concussions caused by different injury events. The main cause of concussion during the Carling Currie Cup 2021 was *Tackling* (27%), followed by being *Tackled* (18%). However, since the *Injury event* dataset was not captured in its entirety for this year, these percentages must be interpreted with caution. Although there are three cases with event *Not provided*, a deep review of the available data was performed. These *Not provided* cases appear to be from *Open play*. Concussions occurring in the *Ruck* have decreased each year from 2019 to 2021. Interestingly, the overall number of concussions recorded remained the same over the last three years.

**Figure 12:**
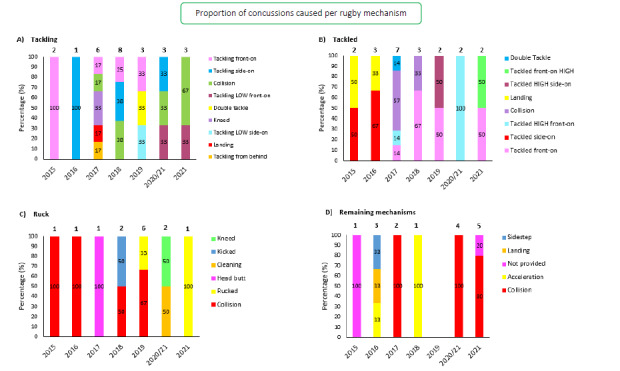
Proportion of concussions caused by A) Tackling, B) Tackled, C) Ruck and D) Remaining concussion mechanisms from 2015 to 2021. (The number above each bar represents the total number of concussions for that event for that year.)

Figure 12 presents the mechanisms contributing to concussions in *Tackling, Tackled, Ruck* and the remaining concussion causing injury events from 2015 – 2021. Data have only been presented from 2015 onwards as *Tackle* related data were not captured separately for the *Tackler* and *Ball Carrier* in 2014.[Fig f27-2078-516x-34-v34i1a15259]

**Figure f27-2078-516x-34-v34i1a15259:**
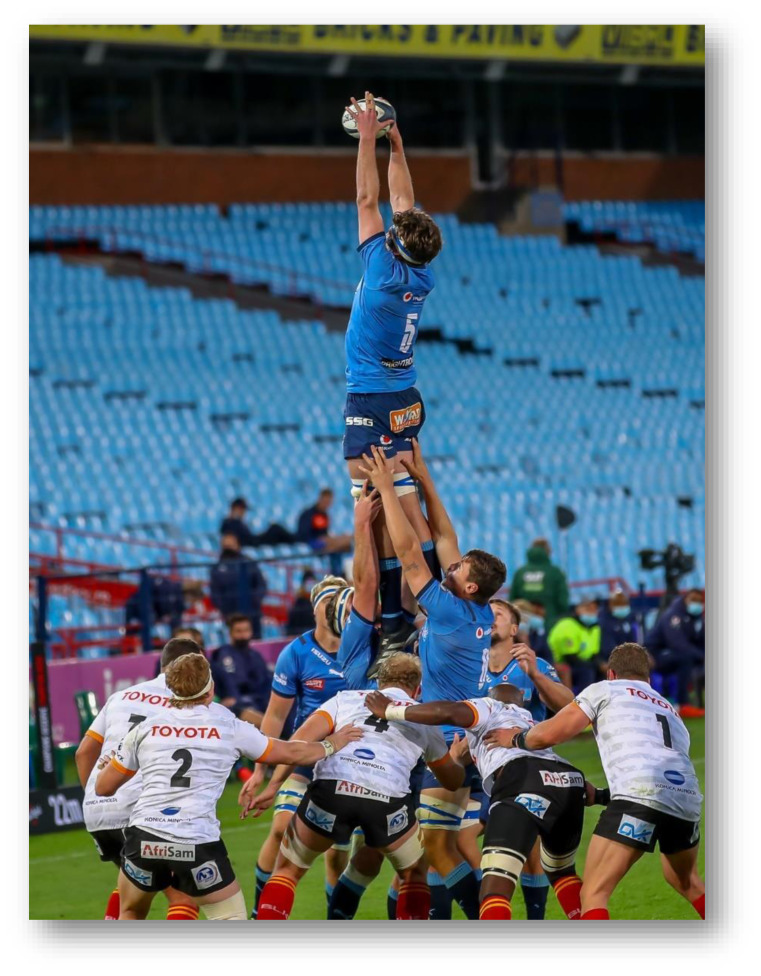


### Region of Injury

The head and knee were the most frequently injured body locations during the Carling Currie Cup 2021 tournament (15% each), followed by the ankle (14%) and shoulder injuries (11%). Concussions (n = 11) contributed to the most head injuries, followed by Lacerations (n = 10). Ligament injuries (n = 12) contributed to the most knee injuries, followed by Bruising/haematoma (n = 5), Osteochondral (n ≤ 3), and Tendon injuries (n ≤ 3). Lastly, Ligament injuries (n = 14) accounted for most of the ankle injuries, followed by unspecified pain (n ≤ 3), tendon injuries (n ≤ 3), synovitis/bursitis (n ≤ 3) and instability (n ≤ 3). The average severity for head injuries was 9 days absent and the injury burden was 117 days absent per 1000 player hours. The ankle injuries had an average severity of 13 days absent, and an injury burden of 169 days absent per 1000 player hours. The knee injuries had the highest average severity of 41 days absent, and an injury burden of 533 days absent per 1000 player hours. The median severity of knee injuries was the highest in the Carling Currie Cup 2021 at 23 days absent. Twenty-five percent of knee injuries resulted in 7 or less days lost from training and matches, and 25% of all knee injuries resulted in 72 or more days lost from training and matches (Table 6).

When analysing the changes in incidence of the most injured body locations for the Carling Currie Cup over the past six seasons, the head and knee remain the first and second most injured body locations from 2017–2021. The injury incidence of head and knee injuries are similar to the 2020/21 season. However, ankle injuries have almost doubled in injury incidence (Table 7).

**Figure 13:**
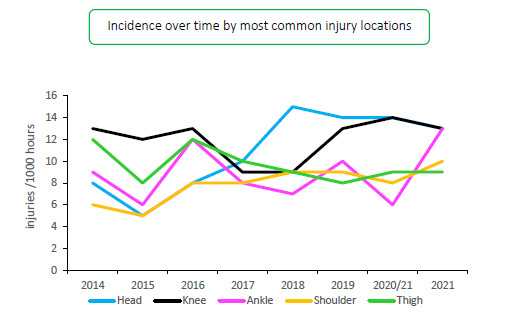
Incidence of the most common injury locations over the surveillance period.

Figure 13 displays the movement of most common injured body locations over the surveillance period (2014–2021). When looking at the injury incidence over the last eight years, there is a noticeable increase in Ankle injuries in 2021. In the 2021 season, ankle injuries reached the highest point in over eight years. Head injuries increased initially from 2015 to 2018, after which they stabilized and are on a slight downward trend since then (Figure 13). This trend links directly to the concussion section earlier in the report, since most head injuries were attributed to concussions.

**Figure 14:**
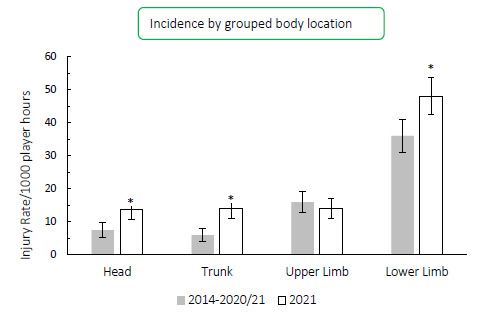
Injury incidence by grouped body location for the Carling Currie Cup 2021 in comparison to the averaged 2014–2020/21 injury rates.

During the Carling Currie Cup 2021, lower limb, head, and trunk injury rates were significantly higher than their 2014–2020/21 average injury rates (Figure 14). The head and knee locations recorded the highest injury rate for the Carling Currie Cup 2021, with injury rates of 13 (7 to 19) injuries per 1000 player hours. The head injury rate was lower than that of the international meta-analysis [1] of 17 (14 to 20) injuries per 1000 player hours and the knee injury rate is similar to the meta-analysis [1] injury rate of 13 (12 to 14) injuries per 1000 player hours.

### Injury Event

In 2021, SARIISPP’s software developers upgraded their data collection software. Unfortunately, the *Injury event* variable could not be captured correctly in this upgraded software package. In some cases, the *Injury event* could be back engineered from the raw data, or was sourced retrospectively from the medical staff, but many cases remained where the medical staff and authors did not want to presume the *Injury event* incorrectly. Therefore, in this report, those *Injury event* cases were recorded as ‘*Not provided*’. As a result, the *Injury event* data, and any changes in *Injury event* data over time for 2021 must be interpreted with caution. This has been addressed with the software developers for future research.

Keeping this in mind, the *Tackle (Ball Carrier)* event accounted for the most injuries in the Carling Currie Cup 2021 (16%, n = 22), followed by the *Ruck* accounting for 12% of injuries (Table 8). When comparing injury rates to the international meta-analysis, *Being tackled* at 15 (8 to 21) injuries per 1000 player hours during the Carling Currie Cup 2021 was slightly lower than the meta-analysis results of 23 (21 to 25) injuries per 1000 player hours but it was not significantly different. *Tackling* during the Carling Currie Cup 2021 at 10 (5 to 15) injuries per 1000 player hours was significantly lower than the meta-analysis of 23 (21 to 25) injuries per 1000 player hours. However, since the *Injury event* data was not captured in its entirety, these data must be interpreted cautiously. *Ruck* injuries during the 2021 season at 11 (5 to 16) injuries per 1000 player hours were marginally higher than the meta-analysis injury rate for the *Ruck* of 9 (7 to 11) injuries per 1000 player hours [1]. Although there are many (36%) *Not provided* cases listed below due to the *Injury event* data capturing issue, 32 (23 to 41) injuries per 1000 player hours, there was a deep review of the raw data, and it seems that most of these *Not provided* cases might be *Open play* related.

**Figure 15:**
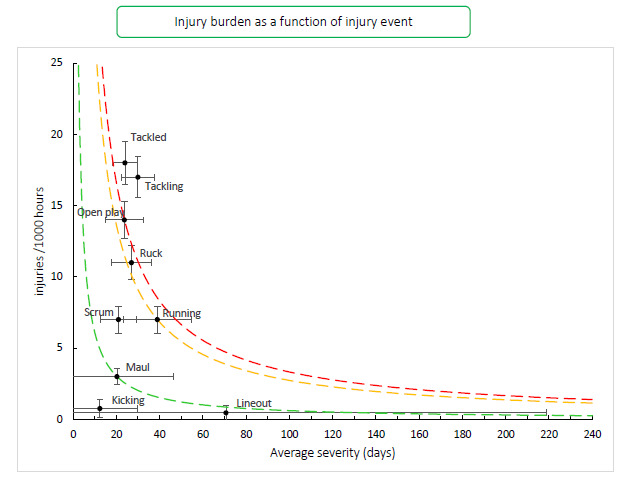
Injury burden as a function of injury event for the seasons 2016 – 2021. The y-axis represents incidence (injuries per 1000 hours), and x-axis represents average severity (days absence). Green line: values to the left and below represent those under the 25th burden percentile; these are low-risk injuries. Orange line: values to the left and below represent those under the 50th burden percentile; these include the low-medium risk injuries. Red line: values to the left and below represent those under the 75th burden percentile; these include the medium-high risk injuries. Values to the right and above the red line are the most high-risk types of injuries, and impact players and teams the most.

Figure 15 combines all the injury types from 2016 – 2021 and presents the injury burden picture over the past six years. Injuries caused by *Tackling* have the highest injury burden for all teams, followed closely by injuries from *Being tackled*. Both these injury events have a high combined injury incidence and average severity. *Open play* and the *Ruck* both followed closely behind these two injury events.[Fig f28-2078-516x-34-v34i1a15259]

**Figure f28-2078-516x-34-v34i1a15259:**
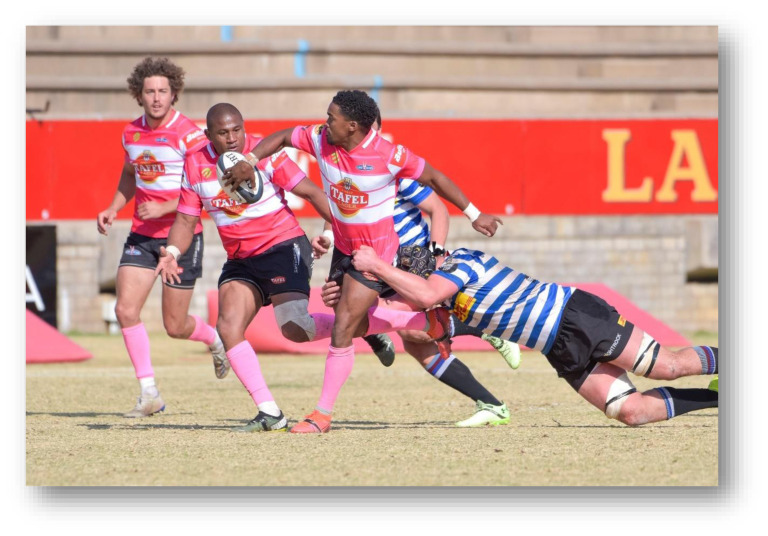


**Figure 16:**
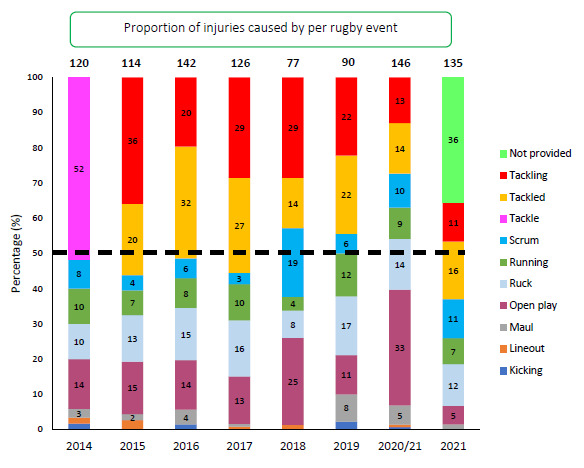
Proportion of injuries caused by the different injury events from 2014 to 2021. (The number above each bar represents the total number of injuries for that year. Tackle data captured separately as tackling and tackled from 2015 onwards).

Figure 16 presents the proportion of injuries caused by injury events from 2014–2021. Over the past six seasons the proportion of injuries caused by *Tackling* have decreased. Only 11% of the injuries were caused by *Tackling* during 2021. However, 2021’s data must be interpreted with some level of caution as explained earlier in this report due to the large number of *Not provided* injury event cases captured in 2021.

### Venue

**Figure 17:**
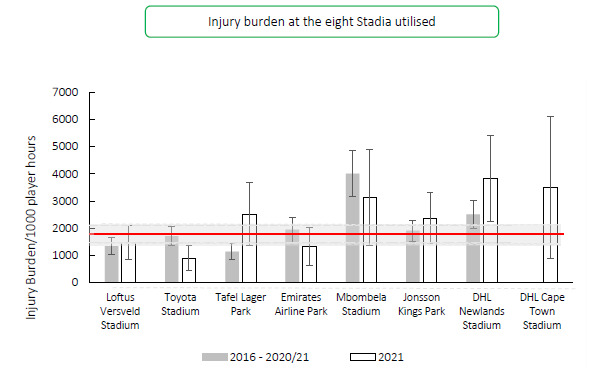
Injury burden/1000 player hours of Time-Loss injuries at the eight utilised stadia in the Carling Currie Cup 2021 in comparison to their averaged 2015–2020/21 injury burden. *Stadium injury burden was significantly higher in 2021 than its 2015–2020/21 average.

Matches were played at eight different stadia during the tournament. This is the first year that the DHL Cape Town Stadium was used during the Carling Currie Cup tournament. DHL Cape Town Stadium’s injury burden is far above the average injury burden and has the second highest injury burden recorded in 2021 (Figure 17).

Table 9 shows the ranking of injury burden of the Stadia from the highest to lowest between 2016–2021. When combining the last six season’s data, it highlights that Mbombela Stadium recorded the highest injury burden overall, with its injury burden being significantly higher than the grouped average injury burden from 2016–2021 (Table 9).

**Figure 18:**
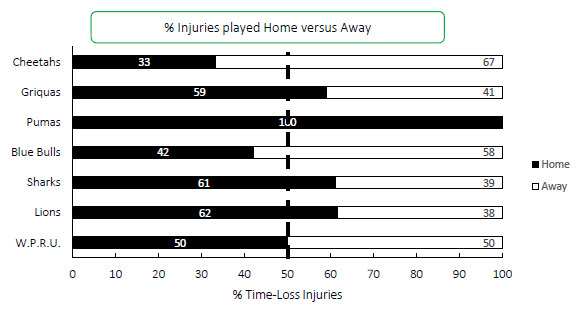
Proportion of injuries sustained playing at home and away venues for the Carling Currie Cup 2021.

Figure 18 presents the proportion of injuries sustained playing at home and away venues at the Carling Currie Cup 2021. When comparing injuries while playing away and at home in the Carling Currie Cup 2021 tournament, playing at home at 47 (32 to 52) injuries per 1000 player hours, recorded a similar injury rate to playing away with 42 (36 to 58) injuries per 1000 player hours. The Airlink Pumas, Cell C Sharks, Sigma Golden Lions and Windhoek Draught Griquas sustained more injuries playing at home than away, while, with the exception of DHL Western Province who were 50:50, the remaining two teams sustained more injuries while playing away.

## TRAINING INJURIES

**Figure 19:**
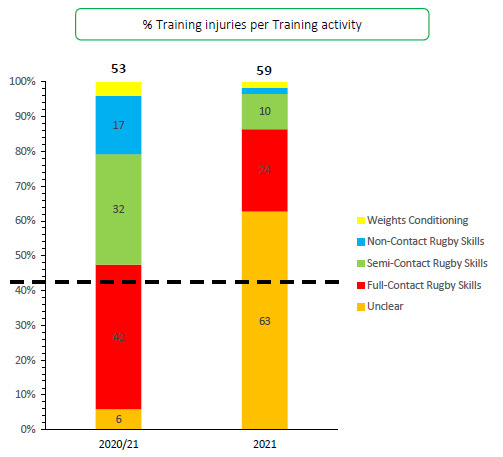
Proportion of Time-Loss training injuries sustained per training activity during the Carling Currie Cup 2020/21–2021.

Overall, 59 Time-loss injuries were sustained during training in the Carling Currie Cup 2021. The time-loss injuries result in an injury incidence of 2 (1 to 2) injuries per 1000 training hours which is lower than the meta-analysis injury incidence of 3 (2 to 4) injuries per 1000 training hours [1]. These contributed to 30% of all injuries experienced during the Carling Currie Cup Tournament over the 2021 rugby season. The average severity of training injury was 42 days, with a median severity (IQR) of 19 (9 to 38) days absent. Figure 19 shows the percentage of training injuries per training activity. In 2021, certain training injuries were not classified into a specific training activity. In some cases, the injuries were sourced retrospectively from the medical staff, but many cases remained where the medical staff and authors did not want to presume in which training activity the injury was classified. Therefore, in this report, those injuries were recorded as ‘*Unclear’*. In future tournaments, these injuries will be followed up more closely to ensure that they are correctly classified.

Table 10 presents the training injuries sustained during the Carling Currie Cup 2021. The most common injury type sustained in all training activities was *Ligament sprain Injuries* carrying the highest average severity at 75 days (Table 10).

The *ankle* was the most injured body location in training accounting for 20% (n = 12) of all Time-Loss training injuries during the Carling Currie Cup 2021, followed closely by the *thigh* and *shoulder* (Table 11).[Fig f29-2078-516x-34-v34i1a15259]

**Figure f29-2078-516x-34-v34i1a15259:**
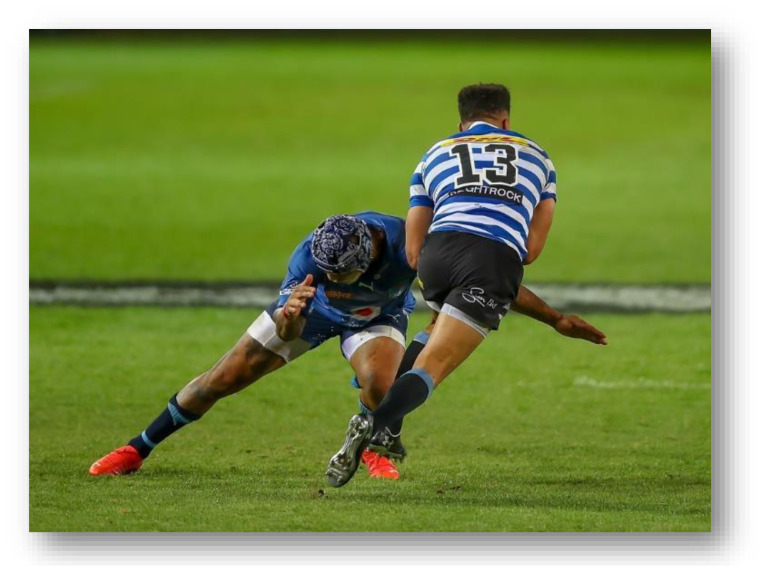


## Figures and Tables

**Figure 1a:**
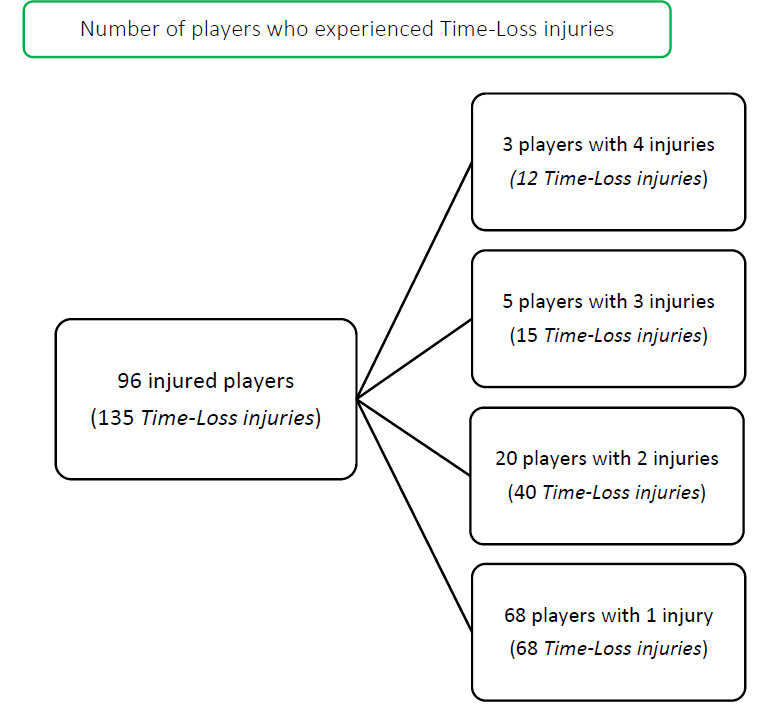
The number of players who experienced Time-Loss injuries during the Carling Currie Cup 2021.

**Figure 1b:**
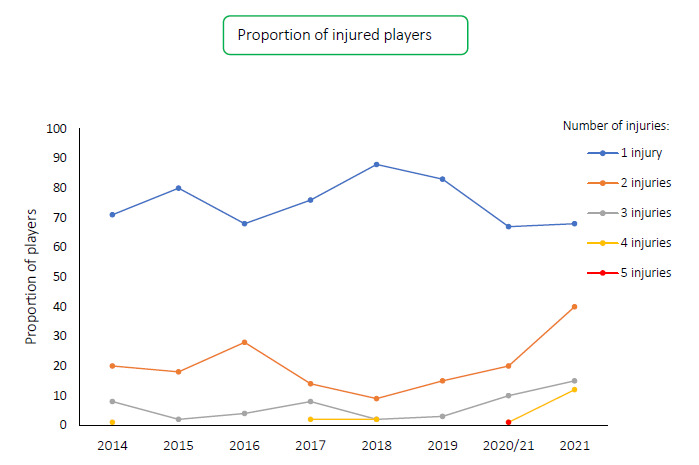
Proportion of injured players experiencing 1 or more injuries in the Carling Currie Cup tournaments from 2014–2021.

**Table 1:** Injury Incidence, Severity (days), Injury Burden (days absent/1000 player hours) and Operational Burden (days absent/injury/match) of Time-Loss injuries for each participating team in the Carling Currie Cup 2021.

Team	Team Injuries/match	Injury Incidence (per 1000 player hours)	Team matches/injury	Total Severity (days lost)	Average Severity (days lost/injury)	Injury Burden (days lost/1000 hours)	Operational Injury Burden (days lost/injury)	Median Severity (IQR)
Vodacom Blue Bulls	1.7	86.4	0.6	453	24	2060	41.2	10 (6 to 34)
Toyota Free State Cheetahs	3.0	150.0	0.3	505	17	2525	50.5	6 (9 to 16)
Sigma Golden Lions	1.3	65.0	0.8	231	18	1155	23.1	12 (4 to 26)
Airlink Pumas	0.3	15.0	3.3	408	136	2040	40.8	143 (124 to 152)
Cell C Sharks	1.8	90.0	0.6	128	7	640	12.8	11 (8 to 17)
DHL Western Province	2.3	115.4	0.4	801	27	3081	61.6	10 (6 to 19)
Windhoek Draught Griquas	1.8	91.7	0.5	415	19	1730	34.6	13 (6 to 24)
***Overall***	***1.8***	***88.8***	***0.6***	***2941***	*22*	*1935*	*38.7*	***10 (6 to 23)***

**Table 2:** The Carling Currie Cup 2021 injuries grouped according to the IOC recommended categories of Tissue and Pathology types for injuries.

Tissue	Incidence	Median time loss	Burden
*Pathology*	Injuries per 1000 hours (95%CI)	Days (95%CI)	*Days per 1000 hours (95%CI)*

**Muscle/Tendon**	**24 (16 to 31)**	**13 (13 to 41)**	**669 (451 to 874)**
Muscle Injury	20 (13 to 27)	12 (11 to 37)	469 (309 to 643)
Tendon rupture	1 (0 to 2)	161	161
Tendinopathy	3 (0.4 to 6)	13 (0 to 53)	86 (10 to 156)
**Ligament/Joint capsule**	**26 (18 to 35)**	**12 (16 to 38)**	**718 (491 to 956)**
Ligament Sprain	24 (16 to 31)	12 (15 to 39)	640 (432 to 837)
Joint Sprain	3 (0 to 5)	30 (10 to 50)	77 (0 to 148)
**Nervous**	**9 (4 to 13)**	**12 (9 to 25)**	**146 (34 to 112)**
Brain/Spinal cord injury	7 (3 to 12)	12 (9 to 21)	108 (45 to 180)
Peripheral nerve injury	1 (0 to 3)	28 (0 to 75)	36 (0 to 84)
**Superficial tissues/skin**	**22 (14 to 29)**	**6 (7 to 15)**	**239 (154 to 319)**
Laceration	7 (3 to 11)	6 (5 to 7)	40 (18 to 66)
Contusion (superficial)	15 (9 to 21)	8 (8 to 18)	196 (117 to 273)
**Bone**	**1 (0 to 3)**	**59 (0 to 138)**	**77 (0 to 177)**
Fracture	1 (0 to 3)	59 (0 to 138)	77 (0 to 177)
**Cartilage/Synovium/Bursa**	**3 (0 to 5)**	**17 (0 to 117)**	**205 (0 to 395)**
Bursitis	1 (0 to 2)	9	9
Cartilage Injury	2 (0 to 4)	23 (0 to 151)	124 (0 to 248)
**Non-specific**	**5 (1 to 8)**	**22 (0 to 76)**	**166 (36 to 288)**

***Overall***	*89 (74 to 104)*	*10 (18 to 30)*	*1935 (1628 to 2288)*

**Table 3:** Proportion (%) of new versus subsequent recurrent injuries for the Carling Currie Cup 2016 – 2021 tournaments.

	2016	2017	2018	2019	2020/21	2021
New injuries	74	74	86	83	68	71
Subsequent recurrent injuries	2.8	3.2	2.6	2.2	3.4	4.4

**Table 4:** Injury rate, Severity and Burden of the most common injury types in the Carling Currie Cup 2021.

Injury Type	Injury Rate (*95%* CI)	Total Severity	Average Severity	Burden (*95%* CI)	Median (IQR)
Sprain Ligament	24 (16 to 31)	868	27	648 (432 to 837)	12 (9 to 19)
Muscle (Rupture/Strain/Tear)	20 (13 to 27)	667	24	480 (312 to 648)	12 (5 to 26)
Contusion/Bruise	15 (9 to 21)	281	13	195 (117 to 273)	8 (6 to 13)
Central Nervous System	7 (3 to 12)	133	15	105 (45 to 180)	12 (8 to 18)
Lacerations	7 (3 to 11)	52	6	42 (18 to 66)	6 (5 to 6)

***Overall***	***88.8 (74 to 104)***	***2941***	***22***	*1935 (1628 to 2288)*	***10 (6 to 23)***

**Table 5:** The movement of the most common OSICS classification diagnoses over the past six seasons [6].

			%	Number	Incidence	Average Severity
2016		Concussion (HN1)	7	10	6 (2–10)	14
		Knee medial collateral ligstr/tear/rupture (KL3)	6	9	6 (2–10)	23
		Hamstring strain/tear (TM1)	6	8	5 (2–9)	11
2017		Concussion (HNCX)	13	16	10 (5–15)	15
		Acromioclavicular jt sprain (SJAX)	10	12	8 (3–12)	25
2018		Concussion (HNCX)	18	14	15 (7–23)	14
		Quadricep strain (TMQX)	5	4	4 (0–8)	18
2019		Concussion (HNCX)	12	11	12 (5–18)	9
		Ankle syndesmosis sprain (AJSX)	5	5	5 (1–10)	14
2020/21		Concussion (HNCX)	8	11	7 (3–11)	10
		Quadriceps haematoma (THV)	4	6	4 (1–7)	4
		Knee strain (MCL)	3	5	3 (0.5–6)	42
2021		Concussion (HNCX)	8	11	7 (3 to 12)	15
		Ankle sprain (AJXX)	6	8	6 (2–10)	10
		Hamstring strain (THHX)	2	5	2 (0–4)	23

**Table 6:** Injury rate, Severity and Burden of the most common injury types in the Carling Currie Cup 2021.

Injury type	Incidence	Total Severity	Average severity	Burden	Median (IQR)
Head	13 (7 to 19)	185	9	117 (63 to 171)	7 (5 to 12)
Knee	13 (7 to 19)	823	41	533 (287 to 779)	23 (7 to 72)
Ankle	13 (7 to 18)	249	13	169 (91 to 234)	9 (5 to 12)
Shoulder	10 (5 to 15)	277	18	180 (90 to 270)	15 (10 to 25)
Thigh Injuries	9 (4 to 14)	301	22	198 (88 to 308)	13 (10 to 24)

***Overall***	***88.8 (74 to 104)***	***2941***	***22***	*1935 (1628 to 2288)*	***10 (6 to 23)***

**Table 7:** The movement of the most injured body locations over the past six seasons.

			%	Number	Incidence	Average Severity
2016		Knee	14	20	13 (7–18)	49
		Ankle	13	18	12 (6–17)	51
		Head	9	13	8 (4–13)	11
		Shoulder	8	12	8 (3–12)	41
2017		Head	13	16	10 (5–15)	15
		Knee	11	14	9 (4–14)	63
		Shoulder	10	12	8 (3–12)	67
		Ankle	10	12	8 (3–12)	87
		A/C Joint	10	12	8 (3–12)	25
2018		Head	18	14	15 (7–23)	18
		Knee	10	8	9 (3–14)	44
		Shoulder	10	8	9 (3–14)	38
		Ankle	9	7	7 (2–13)	65
		Anterior thigh	8	6	6 (1–12)	6
2019		Head	14	13	14 (6 – 21)	8
		Knee	13	12	13 (5 – 20)	13
		Ankle	11	10	10 (4 – 17)	9
		Lower limb posterior	7	6	6 (1 – 11)	3
		Posterior thigh	7	6	6 (1 – 11)	9
2020/21		Head	16	23	14 (9 to 20)	6
		Knee	15	22	14 (8 to 19)	57
		Thigh	10	15	9 (5 to 14)	9
		Shoulder	9	13	8 (4 to 13)	22
		Ankle	7	10	6 (2 to 10)	19
2021		Head	15	20	13 (7 to 19)	9
		Knee	15	20	13 (7 to 19)	41
		Ankle	14	19	13 (7 to 18)	13
		Shoulder	11	15	10 (5 to 15)	18
		Thigh	10	14	9 (4 to 14)	22

**Table 8:** Injury rate, Severity and Burden of the injury events in the Carling Currie Cup 2021

Injury event	Incidence *(95%Cl)*	Total severity	Average severity	Burden	Median (IQR)
Tackle (Ball Carrier)	15 (8 to 21)	363	17	255 (136 to 357)	9 (4 to 32)
Ruck	11 (5 to 16)	578	36	396 (180 to 576)	15 (11 to 61)
Tackle (Tackler)	10 (5 to 15)	269	18	180 (90 to 270)	10 (3 to 33)
Scrum	10 (5 to 15)	512	34	340 (170 to 510)	15 (7 to 61)
Running	7 (3 to 11)	259	26	182 (78 to 286)	16 (7 to 45)
Open play	5 (1 to 8)	123	18	90 (18 to 144)	17 (8 to 27)
Maul	1 (0 to 3)	4	2	2	4
Not provided	32 (23 to 41)	833	17	544 (291 to 697)	10 (9 to 25)
***Overall***	***88.8 (74 to 104)***	***2941***	***22***	*1935 (1628 to 2288)*	***10 (6 to 23)***

**Table 9:** Injury burden/1000 hours of Time-Loss injuries at the eight Stadia utilized in the Carling Currie Cup combined data from 2016 to 2021.

***Stadium***	***Burden (95%Cl)***
***Mbombela Stadium***	*3667 (2941 to 4393)*
*DHL Cape Town Stadium*	*3492 (905 to 6079)*
*DHL Newlands Stadium*	*2845 (2321 to 3370)*
*Jonsson Kings Park*	*2040 (1669 to 2411)*
*Emirates Airline Park*	*1927 (1513 to 2342)*
*Toyota Stadium*	*1645 (1327 to1963)*
*Loftus Versveld Stadium*	*1368 (1094 to 1641)*
*Tafel Lager Park*	*1288 (995 to 1582)*

***Grouped Average***	*1790 (1463 to 2116)*

**Table 10:** Number, average, and median severity of training injuries sustained during the Carling Currie Cup 2021 season according to type of training activity involved.

	Average severity (days)	Median severity (days)
***Rugby skills (full-contact)***	***41***	***20***

Muscle Injury	15	19
Joint injury	16	16
Ligament Sprain	81	81
Fracture	160	160
Concussion		
Nerve Injury	20	20
Tendon Injury	28	28

***Rugby skills (semi-contact)***	***2***	***2***

Muscle Injury	2	2
Ligament Sprain		
Bruising/Haematoma		

***Rugby skills (non-contact)***		

Fracture		

***Conditioning non-weights***		

Unspecified		

***Unclear***	***45***	***17***

Concussion		
Bruising/Haematoma	3	3
Laceration	9	9
Muscle Injury	45	17
Ligament Sprain	75	23
Nerve Injury	10	10
Joint injury		
Synovitis/Capsulitis	10	10
Non-specified	17	17
Tendon Injury	18	6

**Overall**	**42**	**19**

**Table 11:** Number, average, and median severity of training injuries sustained per body location, during the Carling Currie Cup 2021.

	Average severity (days)	Median severity (days)
***Head***	**9**	**9**
***Upper Body***	***32***	***19***

Shoulder	38	19
Upper Arm	2	2
Wrist/Hand	22	22

***Lower Body***	***54***	***23***

Ankle	89	81
Foot	25	25
Hip/Groin	2	2
Knee	15	10
Lower Leg	19	19
Thigh	41	20

***Trunk***	***10***	***10***

Chest	6	6
Lumbar Spine	12	12
Neck	10	10

**Overall**	**42**	**19**
